# Corpus Luteum Color Doppler Ultrasound and Pregnancy Outcome in Buffalo during the Transitional Period

**DOI:** 10.3390/ani10071181

**Published:** 2020-07-13

**Authors:** Luigi Esposito, Angela Salzano, Marco Russo, Donato de Nicola, Alberto Prandi, Bianca Gasparrini, Giuseppe Campanile, Gianluca Neglia

**Affiliations:** 1 Department of Veterinary Medicine and Animal Production, Federico II University, 80137 Naples, Italy; luigespo@unina.it (L.E.); angela.salzano@unina.it (A.S.); denicolavet@gmail.com (D.d.N.); bgasparr@unina.it (B.G.); giucampa@unina.it (G.C.); neglia@unina.it (G.N.); 2 Department of Agricultural, Environmental and Animal Science, University of Udine, 33100 Udine, Italy; alberto.prandi@uniud.it

**Keywords:** buffaloes, color Doppler ultrasound, corpus luteum vascularization, progesterone

## Abstract

**Simple Summary:**

The advent of color Doppler ultrasonography promoted tremendous advances in research and clinical practice in animal reproduction, because it allowed noninvasive visualization of the vascularization in reproductive organs. In particular, the corpus luteum blood flow reflects luteal function better than luteal size in ruminants. Since buffaloes are a seasonal species, it is important to evaluate corpus luteum functionality also during the nonbreeding season, through blood flow examination and early pregnancy diagnosis. For this reason, we selected 29 Mediterranean buffaloes that had undergone synchronization and artificial insemination and were examined daily, from 5 to 10 days post-artificial insemination. Then, we retrospectively classified the buffaloes as pregnant or nonpregnant. Statistical analysis showed that pregnant animals had higher mean progesterone concentration and higher mean time average medium velocity values from Day 5 to Day 10 compared to nonpregnant buffaloes. Moreover, these two parameters could be used to predict the likelihood of pregnancy, starting on Day 6, although more reliable results could be obtained at Day 10 post-artificial insemination. In conclusion, a proper CL growth and development from Day 5 to Day 10 post-timed artificial insemination (TAI) is crucial for pregnancy maintenance during the transitional period.

**Abstract:**

This study evaluated corpus luteum (CL) development in buffaloes out of breeding season and assessed an early pregnancy diagnosis. Mediterranean buffaloes (n = 29) were synchronized and artificially inseminated. CL B-mode/color Doppler ultrasonography examinations were performed daily from Days 5 to 10 post-synchronization, recording CL dimensions and blood flow parameters. Blood samples were collected on the same days for the progesterone (P_4_) assay. Data were grouped into pregnant or nonpregnant and retrospectively analyzed. The total pregnancy rate was 50.0% (13/26) on Day 45. A significant difference between CL average area in pregnant and nonpregnant buffaloes was recorded only on Day 10. Pregnant buffaloes showed a significantly higher mean P_4_ concentration and higher mean time average medium velocity (TAMV) values from Day 5 to Day 10 compared to nonpregnant buffaloes. Linear regression analysis showed a significant relationship between P_4_ levels and TAMV. Multiple logistic regression highlighted a significant influence of TAMV on pregnancy outcome, particularly on Day 8. This is probably due to the strong relationship between TAMV and P_4_ production. Both TAMV and P_4_ could be used to predict pregnancy starting on Day 6, although a more reliable result was obtained at Day 10. Thus, the period between Days 5 and 10 is critical for CL development during the transitional period in buffalo.

## 1. Introduction

The growing demand for meat and milk worldwide has caused a steady increase in the buffalo population in the last few years in both developed [1] and developing countries [2,3]. Buffaloes show high adaptability to different climates and conditions, and can be considered to be an important source of animal-derived protein for human consumption [1]. Although breeding is often managed on small farms, representing the basal rural economy in Asia, intensive breeding systems are performed in other countries such as Italy [4]. However, its productive efficiency is strongly related to the reproductive pattern, which is influenced by the photoperiod. Indeed, this species is considered to be a short-day breeder, meaning that it increases its reproductive activity when daylight hours decrease [4]. In Italy, these reproductive characteristics are opposite of the market requirement for milk production, suggesting that the application of the out-of-breeding season mating technique is responsible for a decline in reproductive efficiency, particularly during some periods of the year [4]. Embryonic mortality represents one of the main factors that are involved in pregnancy failure in both buffalo [5,6] and bovine [7] species. Several studies demonstrated that early development of the corpus luteum (CL) together with high progesterone (P_4_) levels are involved in adequate embryonic growth and reduction of embryonic mortality [8,9]. In particular, a study performed during the breeding season showed that successfully pregnant buffaloes had larger CL development, higher P_4_ concentrations, and increased blood flow to the CL, measured as the time average medium velocity (TAMV), compared to nonpregnant counterparts [10]. The latter experiment was performed during the breeding season from Day 5 to Day 10 post-timed artificial insemination (TAI). Furthermore, a low pregnancy rate was associated with delayed vascularization of the CL after Day 5. However, it cannot be ruled out that CL development occurs differently during the transitional period from mid-winter to spring when the females show a decrease in reproductive activity in response to increasing day length. The incidence of embryonic mortality during this period increases to 40% on some farms [11,12]. Therefore, the aims of this study were to: (i) evaluate CL development during the transition to the seasonal nadir in reproductive activity (transitional period from decreasing to increasing daylight length) through blood flow examination; (ii) determine whether CL blood flow parameters or progesterone levels can be used to estimate the likelihood of pregnancy by using the receiver operating characteristics (ROC) analysis, even if very early during CL development.

## 2. Materials and Methods

### 2.1. Animals

The Ethical Animal Care and Use Committee of the Federico II University of Naples approved the experimental design. The trial was performed in February 2018 on 29 pluriparous Italian Mediterranean buffaloes that were 120.2 ± 18.3 days in milk and bred on a commercial farm in the South of Italy between 40.5° N and 41.5° N parallel. The animals were maintained in open yards that allowed 15 m^2^ per animal and 80 cm manger space. The buffaloes were fed a total mixed ration consisting of 55% forage and 45% concentrate, containing 0.91 milk forage units/kg of dry matter and 15% crude protein. To assess the cyclic status, all the animals underwent two ultrasound examinations, spaced 12 days apart (MyLab 30Gold, Esaote, Genova, Italy) using a 7.5 MHz transrectal linear probe, before the start of the synchronization protocol. Only animals with a functional CL in at least one examination were included in the study.

### 2.2. Synchronization of Ovulation and Timed Artificial Insemination

Buffaloes were synchronized using the Ovsynch TAI program, which was developed for cattle [13] and is widely used in buffalo [14]. Briefly, 20 µg of gonadotropin-releasing hormone (GnRH) analogue (Buserelin acetate, 12 μg; Receptal^®^, Intervet, Milan, Italy) was administered on Day 0 followed by a synthetic analogue of prostaglandin F2α (Cloprostenol^®^, 0.250 mg/mL MSD, Milan, Italy) on Day 7 and an additional GnRH analogue administration on Day 9, with TAI on Day 10. All the animals underwent ultrasound examination on Day 10 of the synchronization protocol. Only those with a follicle that was larger than 1 cm and with a tonic uterus, with or without mucus vaginal discharge, were inseminated by the same experienced operator 20 h after the second GnRH injection. The dimensions of the preovulatory follicle were recorded on the day of TAI, and the follicle area was calculated according to the following equation:

FL area = (a/2) × (b/2) × π(1)

where:

✓ FL = follicle
✓ a = major axis of the follicle
✓ b = minor axis of the follicle

Furthermore, ultrasound examinations were made one day post-AI to be sure that all the animals underwent ovulation.

### 2.3. Corpus Luteum and Blood Flow Evaluation

**Figure 1:**
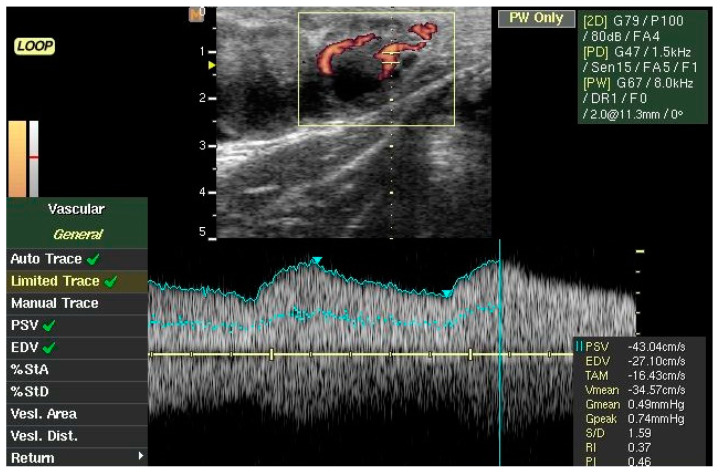
Corpus luteum blood flow assessment in buffaloes with the aid of eco-color Doppler mode.

CL ultrasonography examinations were performed daily from Day 5 to Day 10 post-TAI using a portable ultrasound unit (MyLab 30Gold, Esaote, Genova, Italy) that was equipped with a 7.5 MHz linear transducer, designed for transrectal examination in large animals (LV 513). To obtain a better definition of the CL, once the ovary was visualized, the image was adjusted and then frozen to measure both the long and short axes. The CL area was calculated as follows: CL area = (a/2) × (b/2) × π, where a is the long axis; b is the short axis. To assess blood flow characteristics, the color Doppler mode was activated in order to evaluate the corpus luteum blood flow and, when the CL was clearly visualized, the sample gate was placed at the level of the primary branch of the *ramus tubarius* of the ovarian artery, just before hugging the corpus luteum. At this level, the artery was reasonably accessible and sufficiently large (2 to 3 mm in diameter) in order to measure flow rates accurately and in particular the flow indexes (TAMV, Resistivity Index (PI) and Pulsatility Index (PI); Figure 1). Color gain setting, velocity setting, and a color-flow filter setting were standardized during all the procedures and all the analyses were carried out by the same technician. In particular, the pulse repetition frequency was set at 0.7 kHz, the lowest possible that did not result in aliasing artifact, the power Doppler frequency was set to 1.5 kHz with a gain of 47, and the pulsed wave Doppler was set at 8 kHz, gain 67. Real-time B-mode/Color Doppler images of the continuous scans of the CL were recorded and then analyzed retrospectively. B-mode and color-flow mode short video clips (7 s duration) were stored in the internal memory of the ultrasound machine. Later, as described by Siqueira et al. [15], the CL area and the area of the cavity (where present) were assessed using the machine’s internal calipers. To justify fluid-filled cavities, CLS was assessed by subtracting the area of the cavity from the entire CL area.

### 2.4. Progesterone Assay

Blood P_4_ levels were assessed using a radioimmunoassay (RIA) according to [16] to determine CL functionality. All the analyses were performed in the Laboratory of Veterinary Physiology of the University of Udine. Blood samples were collected from the jugular vein into heparinized tubes on the same days as ultrasound examination (from Day 5 to Day 10). The samples were centrifuged at 800× *g* for 15 min and the plasma was stored at −20 °C until it was required for the P_4_ assay, which was performed at the same time for all samples. The minimum detectable amount of P_4_ was 2.2 ± 0.07 pg and the intra-assay and inter-assay coefficients of variation were 6.4% and 12.1%, respectively. Values of 1.2 ng/mL were considered to indicate the presence of a functional CL, according to previous studies [10].

### 2.5. Pregnancy Diagnosis

Pregnancy diagnosis was performed on Day 27 post-TAI using the same ultrasound machine as described above. Pregnancy status was confirmed on Day 45 and 70 post-TAI. Animals that were pregnant on Day 27 but not pregnant on Day 45 were considered to have encountered late embryonic mortality (LEM). Similarly, those that were pregnant on Day 45 and not pregnant on Day 70 were considered to have undergone fetal mortality.

### 2.6. Statistical Analysis

A repeated measure ANOVA was performed to evaluate the differences between pregnant and nonpregnant animals for P_4_ levels, CL area, TAMV, PI, and RI values that were recorded from Day 5 to Day 10 [17]. Because of the low number of animals, the latter analysis was confirmed by another repeated measure ANOVA using a nonparametric approach [18,19]. Chi-square analysis was performed to evaluate differences in the pregnancy rate between buffaloes that showed an early or delayed increase in P_4_. A linear regression model was applied to evaluate the effects of TAMV, RI, PI, and CL area on P_4_ blood levels, either on each day or throughout the experimental period. Moreover, a multiple logistic regression assay was performed to calculate the odds ratios for pregnancy using the TAMV, RI, PI, and CL area on P_4_ blood levels as dependent variables [17]. All data were expressed as the mean ± standard error of the mean (SEM). Additionally, receiver operating characteristics (ROC) analyses were performed, focusing on CL characteristics (CL area, RI, PI, TAMV, and P_4_ concentrations) on Day 5 to Day 10 post-TAI to identify the optimal cutoff value for predicting pregnancy. The latter was determined from the data point that minimized the distance and reported the relative sensitivity and specificity percentages and area under the curve (AUC) value.

## 3. Results

### 3.1. Pregnancy Rate

Total pregnancy rate on Day 27 was 57.7% (15/26), and it was reduced to 50.0% on Day 45 after TAI. This means that the incidence of embryonic mortality was 13.3% (2/15). For the subsequent analysis, buffaloes that underwent LEM were included in the nonpregnant group. No fetal mortality was recorded. No differences were recorded between follicular area in both groups (data not shown). Average CL area was similar from Day 5 to Day 10 in pregnant animals compared to their nonpregnant counterparts (Table 1), although a (*p* < 0.05) difference was recorded on Day 10. Pregnant buffaloes also showed higher (*p* < 0.01) P_4_ concentration for the Day 5 to Day 10 period compared to nonpregnant buffaloes, although significant differences were recorded from Day 7 on. The P_4_ levels that were higher than 1.2 ng/mL on Day 5 post-TAI were recorded in 42.3% of buffaloes (11/26) and 81.8% (9/11) of them were pregnant. In 69.2% (9/13) of the pregnant buffaloes, P_4_ concentrations that were higher than 1.2 ng/mL were recorded, whereas 30.8% (4/13) showed lower P_4_ concentrations on Day 5 post-TAI. No effect of the day on pregnancy and no interactions were found with the repeated measures ANOVA.

### 3.2. Corpus Luteum Characteristics

**Figure 2:**
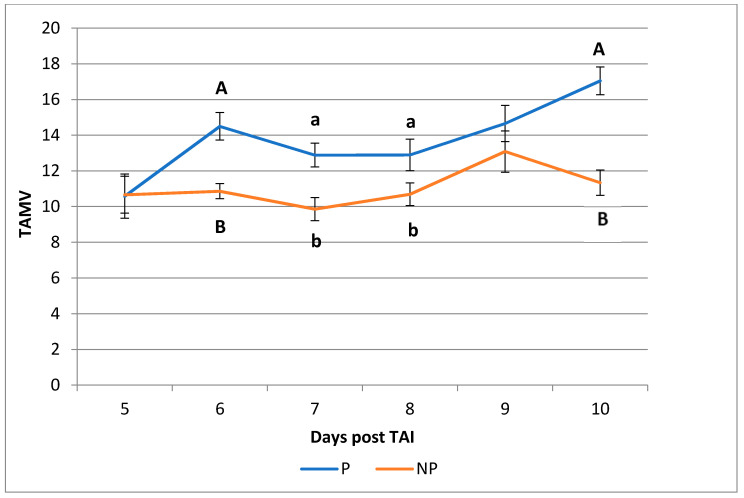
Time average medium velocity (TAMV) values recorded in pregnant (P) and nonpregnant (NP) buffaloes from Day 5 to Day 10 post-timed artificial insemination (TAI). ^A, B^ Values with different superscripts are significantly different; *p* < 0.01; ^a, b^ Values with different superscripts are significantly different; *p* < 0.05.

No differences were recorded for the RI (0.41 ± 0.0 vs. 0.41 ± 0.0, in pregnant and nonpregnant buffaloes, respectively) and the PI (0.56 ± 0.0 vs. 0.55 ± 0.0, in pregnant and nonpregnant buffaloes, respectively) values, either on single days or throughout the experimental period. However, the mean TAMV value from Day 5 to Day 10 was (*p* < 0.01) higher in pregnant compared to nonpregnant buffaloes (14.05 ± 0.45 vs. 10.39 ± 0.57 cm/s), and significant differences were present from Day 6 (Figure 2).

The characteristics of the CL blood flow that were evaluated by the eco-color Doppler technique showed a delayed vascularization in six buffaloes (19.2%). Blood flow features (TAMV, RI, PI) on Day 5 post-TAI were not recorded in these animals. Among these animals, only one buffalo (17.0%) was diagnosed as pregnant, whereas a higher pregnancy rate (60.0%; 12/20; *p* < 0.05) was recorded in buffaloes that showed a competent blood flow on Day 5 post-TAI.

### 3.3. Regression Analysis

The multiple linear regression analysis, considering the average of all parameters between Day 5 and Day 10, showed a significant relationship (R^2^ = 0.394; *p* < 0.05) between P_4_ levels and TAMV. The CL area tended (*p* = 0.096) to influence P_4_ levels, according to the following equation:

P_4_ = 0.287 + (0.263 × CL Area) + (0.081 × TAMV) (2)

The same analysis was performed each day. In this case, the influence of TAMV and the CL area on P_4_ levels was recorded only on Day 8 (R^2^ = 0.303; *p* <0.05), according to the following equation:

P_4_ = 0.117 + (0.334 × CL Area) + (0.082 × TAMV) (3)

The multiple regression analysis in pregnancy that was performed using the average values from the period Day 5 to Day 10 of P_4_, CL area, TAMV, RI, and PI showed that pregnancy outcome was significantly influenced only by TAMV (odds ratio = 3.808; *p* < 0.01). If the multiple regression analysis was performed considering the same parameters on each day, a significant influence of TAMV was recorded also on Day 6 (odds ratio = 2.581), Day 7 (odds ratio = 1.899), and especially on Day 8 (odds ratio = 10.325).

### 3.4. Predicting Pregnancy

The ROC analyses indicated that both TAMV and P_4_ were useful to predict pregnancy compared to other endpoints starting on Day 6 (Table 2 and Table 3). Based on the distance from the ideal point, our analysis defined the best TAMV cutoff value as 11.88 cm/s for pregnancy prediction on Day 6 (sensitivity 76.9% and specificity 76.9%; *p* < 0.01). The P_4_ cutoff value was 1.14 ng/mL for the pregnancy prediction on Day 6 (sensitivity 84.6% and specificity 61.5%; *p* < 0.05). However, Day 10 after TAI showed the best results for TAMV and P_4_ in terms of sensitivity and specificity. The TAMV cutoff value was set at 13.19 cm/s, with an increase in sensitivity (92.3%) and specificity (84.6%; *p* < 0.01), while the P_4_ cutoff value was set at 1.69 ng/mL, with an increase in sensitivity (92.3%) and specificity (92.3%; *p* < 0.01). For the other parameters, it was possible to set a cutoff value (2.38 cm^2^) for the CL area only beginning on Day 10, with a sensitivity of 76.9% and a specificity of 53.8% (*p* < 0.05). It was not possible to set a cutoff value for RI and PI.

## 4. Discussion

This study analyzes the detailed CL development from day 5 to day 10 post-TAI in buffalo species during the transitional period to the seasonal nadir in reproductive activity. Although some studies of this type were performed [10], CL growth and characteristics were only investigated during the breeding season. Thus, we based our experimental design on that paper and we performed this study to confirm the results obtained by Neglia et al. [10] during the buffalo nonbreeding season. The CL is known to play a pivotal role in pregnancy establishment in several mammalian species, because of its capability to secrete P_4_ [20]. The sensitivity to decreasing daylight hours deeply affects the reproductive efficiency of the buffalo, as demonstrated by the reduced CL functionality in some periods of the year [21,22]. This is responsible for the decline in reproductive efficiency and the increase in embryonic mortality [6].

The CL functionality can be assessed using several tools. The CL ecotexture that was assessed by ultrasound was one of the first approaches that was used to evaluate the CL [23,24], and the main limit of this technique was its subjective determination, which does not allow large-scale utilization. However, two reliable indicators of CL functionality are P_4_ blood levels and/or the CL blood flow [25]. In our study, higher P_4_ levels were recorded in pregnant buffaloes during the Day 7 to Day 10 period, which can be considered to be a critical window for embryonic growth and development. These results are in agreement with previous trials that were performed during the breeding season [10,25], in which the same trend was observed. High P_4_ levels, and particularly an early increase in P_4_ levels during the first days after mating, is responsible for larger embryonic development, high interferon-tau levels, and, consequently, reduced embryonic mortality, which represents one of the main causes of reproductive failure in buffalo [6,9,26]. This is also demonstrated by the evidence that animals that ovulated after treatment with a GnRH agonist on Day 5 after TAI showed an early increase in the P_4_ concentration and a high pregnancy rate [11]. In our trial, significantly higher P_4_ levels between pregnant and nonpregnant buffaloes were recorded starting from Day 7 and were definitely higher from Day 8. This interesting aspect further confirms the hypothesis that this period is critical for embryonic development, as previously observed [10]. Furthermore, more than 80% of buffaloes that showed P_4_ levels that were higher than 1.2 ng/mL on Day 5 post-TAI were diagnosed as pregnant on Day 45, which strongly supports the evidence that an early increase in P_4_ levels is fundamental for pregnancy outcome. It is still unclear if the CL dimensions may be considered to be a reliable indicator for P_4_ production and/or the pregnancy rate.

Although CL area tended to be larger in pregnant buffaloes starting from Day 8 after TAI, a significant difference was only observed on Day 10 and similar values were recorded on average during the Day 5 to Day 10 period. The influence of CL dimensions on pregnancy rate is controversial. While in some studies, pregnant buffaloes showed a higher CL area compared to nonpregnant subjects [10,27], no differences were observed in other studies [28,29]. In a recent trial [30], a difference in CL dimensions between pregnant and nonpregnant buffaloes was observed only 14 days after insemination, while no differences were recorded during the Day 5 to Day 10 period. Therefore, further investigations are needed to clarify this aspect. Conversely, according to other authors, CL blood flow can be considered to be a consistent indicator of luteal functionality rather than its dimensions [31]. The results obtained in our trial further confirm this interesting aspect because TAMV values significantly affect the likelihood of pregnancy, as observed in previous studies [10]. After ovulation, the developing CL needs blood substrates for P_4_ production and several studies support a strong correlation between blood flow and P_4_ levels, either in cattle [32] or in buffalo [10]. However, a significant increase of blood flow has been correlated with pregnancy in cattle only 15 days after insemination [33], although some recent studies performed in Holstein cows highlight a difference between pregnant and nonpregnant cattle from Day 7 post-estrus [28]. In the present trial, TAMV values recorded on Day 8 post-TAI seem to significantly influence the likelihood of pregnancy in the buffalo species. This was supported by a recent study in which a significantly higher luteal blood flow and TAMV values were observed on Day 7 post-estrus in successfully pregnant Holstein cows, which were used as recipients for ET and treated with GnRH on Day 5, compared to nonpregnant counterparts [28]. However, according to these authors, the simultaneous evaluation of TAMV and the blood flow area may increase the accuracy of pregnancy prediction in cattle. In our trial, the blood flow area was not measured, but a significant influence of TAMV values was observed on Day 8, rather than Day 7. However, a delayed evaluation of blood flow on Day 8 cannot be ruled out, which may also increase the chances of pregnancy prediction in cattle. If these interesting findings are confirmed, this parameter may be used for an early pregnancy diagnosis either in cattle or in buffalo.

To determine the reliability of our results and to use them in a practical way, we used the ROC curve [34] to set a good cutoff value to predict pregnancy. Our results confirmed that both TAMV and P_4_ blood levels at Day 6 could already provide some important information, but more reliable results in terms of sensitivity and specificity could be recorded directly on Day 10. Kanazawa et al. [29] made a similar experiment in cattle and reported that already at Day 7 it was possible to set up a cutoff value for both TAMV and P_4._ Considering these findings, both TAMV and P_4_ measurements seem to be better indexes of luteal function compared to the CL area. Moreover, the use of color Doppler ultrasonography could avoid stressful conditions for an animal that could not undergo blood sampling. We are aware that, very early during the CL development, we had cutoff values with low sensitivity and specificity. In our study, the highest values for sensibility and specificity among the days evaluated (5 to 10) were observed on Day 10. Actually, Day 10 may not be the best day to perform a nonpregnancy diagnosis based on CL vascularization, in the perspective of practical use. However, postponing this specific time window will delay the resynchronization of the open females and allow breeders to save money. Moreover, there will always be false positives, such as animals that, due to asynchronized ovulations, fail to respond to the TAI or that undergo embryo mortality. We are aware that further studies and a higher number of animals are necessary to confirm our results.

## 5. Conclusions

In conclusion, this study demonstrated that proper CL functionality and development from Day 5 to Day 10 post-TAI are crucial for pregnancy maintenance. The evaluation of CL blood flow using an eco-color Doppler technique is a noninvasive procedure that can be practically used to obtain useful information on CL functionality. Finally, the evaluation of the TAMV in the CL from Day 8 after TAI can be used to estimate likelihood of pregnancy, and thus to estimate the potential benefits of using an early resynchronization strategy to increase the number of pregnancies in buffaloes.

## Figures and Tables

**Table 1:** Corpus luteum (CL) area and circulating concentrations of progesterone (P_4_) from Days 5 to 10 in buffaloes that were subsequently diagnosed as pregnant (P) or nonpregnant (NP).

Days	CL Area (cm^2^)		P_4_ (ng/mL)	
	P (n = 13)	NP (n = 13)	P (n = 13)	NP (n = 13)
5	1.80 ± 0.1	1.72 ± 0.2	1.24 ± 0.1	1.11 ± 0.1
6	1.86 ± 0.2	1.84 ± 0.2	1.45 ± 0.1	1.26 ± 0.2
7	2.32 ± 0.2	2.10 ± 0.2	1.70 ± 0.1 ^a^	1.32 ± 0.1 ^b^
8	2.49 ± 0.2	2.14 ± 0.1	1.91 ± 0.1 ^A^	1.25 ± 0.1 ^B^
9	2.70 ± 0.2	2.25 ± 0.2	2.09 ± 0.1 ^A^	1.32 ± 0.2 ^B^
10	2.66 ± 0.1 ^a^	2.23 ± 0.1 ^b^	2.28 ± 0.1 ^A^	1.36 ± 0.1 ^B^
Day 5 to Day 10	2.31 ± 0.1	2.05 ± 0.1	1.78 ± 0.1 ^A^	1.27 ± 0.1 ^B^

Values are expressed as mean ± standard error. ^a,b, A,B^: For each endpoint, values with different superscripts within the same row differ. ^a,b^: *p* < 0.05; ^A,B^: *p* < 0.01.

**Table 2:** Summary of the ROC analyses of the time average medium velocity (TAMV) from Day 6 to Day 10 post TAI in buffaloes during the transitional period.

Day	Items	Cutoff Value	AUC Value	Sensitivity (%)	Specificity (%)	*p* Value
6	TAMV	11.88 cm/s	0.90	76.9	76.9	*p* < 0.01
7	TAMV	10.53 cm/s	0.87	84.6	69.2	*p* < 0.01
8	TAMV	11.00 cm/s	0.82	84.6	61.5	*p* < 0.01
9	TAMV	12.13 cm/s	0.75	69.2	69.2	*p* < 0.05
10	TAMV	13.19 cm/s	0.94	92.3	84.6	*p* < 0.01

ROC, receiver operating characteristics; AUC, area under the curve.

**Table 3:** Summary of the ROC analyses of the progesterone (P_4_) levels from Day 6 to Day 10 post-TAI in buffaloes during the transitional period.

Day	Items	Cutoff Value	AUC Value	Sensitivity (%)	Specificity (%)	*p* Value
6	P_4_	1.15 ng/mL	0.73	84.6	61.5	*p* < 0.05
7	P_4_	1.34 ng/mL	0.73	84.6	61.5	*p* < 0.05
8	P_4_	1.47 ng/mL	0.89	92.3	76.9	*p* < 0.01
9	P_4_	1.60 ng/mL	0.88	92.3	69.2	*p* < 0.01
10	P_4_	1.69 ng/mL	0.95	92.3	92.3	*p* < 0.01

ROC, receiver operating characteristics; AUC, area under the curve.
